# A Novel Cellular Senescence Gene, *SENEX*, Is Involved in Peripheral Regulatory T Cells Accumulation in Aged Urinary Bladder Cancer

**DOI:** 10.1371/journal.pone.0087774

**Published:** 2014-02-05

**Authors:** Tianping Chen, Huiping Wang, Zhiqiang Zhang, Qing Li, Kaili Yan, Qianshan Tao, Qianling Ye, Shudao Xiong, Yiping Wang, Zhimin Zhai

**Affiliations:** 1 Department of Hematology, Second Hospital of Anhui Medical University, Hefei, China; 2 Hematology Research Centre, Second Hospital of Anhui Medical University, Hefei, China; 3 Department of Urology, Second Hospital of Anhui Medical University, Hefei, China; 4 Central Laboratory, Anhui Provincial Hospital, Hefei, China; 5 National Institutes of Health and WESTMEAD Transplants and Kidney Disease Research Centre, Sydney University, Sydney, Australia; Rutgers - New Jersey Medical School, United States of America

## Abstract

Regulatory T cells (Tregs) play an essential role in sustaining self-tolerance and immune homeostasis. Despite many studies on the correlation between Tregs accumulation and age, or malignancies, the related mechanism hasn’t been well explored. To find out the mechanism of Tregs accumulation in aged urinary bladder cancer, we examined the novel cellular senesence gene *SENEX* and relevant apoptosis gene mRNA expression in sorted CD4+CD25^hi^ Tregs from aged UBC donors, evaluated serum cytokine profiles related to tumor immunopathology, and further explored the relationship between *SENEX* expression, apoptosis gene expression and cytokine secretion. After having silenced down SENEX gene expression with RNA interference, we also evaluated the cellular apoptosis of Tregs sorted from aged UBC patients in response to H_2_O_2_-mediated stress. Our data indicated that upregulated *SENEX* mRNA expression in Tregs of aged UBC patients was correlated with pro-apoptotic gene expression and cytokine concentration. Silencing *SENEX* gene expression increased cellular apoptosis and pro-apoptotic gene expression of Tregs, in response to H_2_O_2_-mediated stress. Upregulated *SENEX* mRNA expression together with decreased pro-apoptotic gene expression and disturbances in cytokines synthesis may contribute to the Tregs proliferation and promote tumorigenesis and metastasis. Overall, upregulation of cellular senescence gene *SENEX*, was associated to regulatory T cells accumulation in aged urinary bladder cancer. Our study provides a new insight into understanding of peripheral Tregs accumulation in aged malignancies.

## Introduction

Regulatory T cells (Tregs) play an essential role in sustaining self-tolerance and immune homeostasis by suppressing a wide variety of physiological and pathological immune responses against self and nonself, as well as quasi-self tumor antigens [1], [2]. Recruited to tumor tissues, Tregs expand and become activated in tumor tissues and in the draining lymph nodes, suppress the antitumor immune responses [3]. It is found that peripheral blood CD4+CD25+Tregs accumulate dramatically in healthy aged humans and rodents [4]–[5]. The increase in Tregs proportion contributes to age-related immune-suppression, increases susceptibility to infectious diseases and cancer [6]. Further, increased numbers of Tregs have been observed in patients with urinary bladder cancer (UBC), with tumor expression of FoxP3, a specific Tregs marker, correlated with clinical prognosis [7]. Intratumoral accumulation of Tregs have also been observed in colorectal cancer, gliomas and hepatocellular carcinoma [8]–[10]. Suppressing the anti-tumor response, these accumulated intratumoral Tregs are found to be related to poor prognosis[8]–[10]. Despite all the above research endeavors, the detailed mechanisms haven’t been well elucidated. A recent study approaches Tregs accumulation from a new perspective, reporting that cellular apoptosis is involved in Tregs accumulation in aging process. After 24h in culture, Tregs from old animals died significantly less over the course of this assay than did Tregs from young mice, suggesting that Tregs from aged mice are more resistant to apoptosis [11]. However, the mechanisms underlying their resistance to apoptosis in aged humans, especially in aged cancer patients are yet to be explored.

Recent studies have illustrated that upregulated cellular senesence may contribute to the apoptosis resistance of senescent human cells. An investigation has made the identification of a novel gene, *SENEX*, that regulates stress induced premature senescence pathways (SIPS) in endothelial cells (ECs) [12]. Interestingly, these senescent cells induced by *SENEX* are resistant to tumor necrosis factor (TNF-α) induced apoptosis [12]. Furthermore, under certain circumstances, some senescent cells could even change their phenotypes into opposite lineages. Novel mechanism reveals that human Tregs could induce senescence but not apoptosis in responder naïve and effector T cells [13]. These senescent responder T cells induced by Tregs change their phenotypes and cytokine profiles, and possess potent suppressive function. Great attention has been recently paid to cellular senescence, however, the potential role of senescence gene expression in aged cancer patients involved in Tregs accumulation remains largely unknown.

To analyze the potential contribution of senescence and apoptosis gene expression to peripheral Tregs accumulation in aged malignancies, we firstly sorted CD4+CD25^hi^ Tregs in aged UBC patients, and then examined the transcriptional levels of relevant gene mRNA expression in sorted CD4+CD25^hi^ Tregs. Furthermore, serum cytokine profiles related to tumor immunopathology were evaluated as indirect assessment for T cell function. We also assessed the relationship between senescence gene *SENEX* mRNA expression and cytokine secretion. Then, we evaluated the cellular apoptosis of short-term cultured Tregs from aged UBC patients in response to H_2_O_2_-mediated stress after *SENEX* gene expression was silenced with RNA interference. Our data provided evidence that upregulated *SENEX* mRNA expression in Tregs of aged UBC patients were correlated with cytokine concentration and pro-apoptotic gene expression. Silencing *SENEX* gene expression increased cellular apoptosis and pro-apoptotic gene expression in short-term cultured Tregs, in response to H_2_O_2_-mediated stress. Upregulated *SENEX* mRNA expression together with decreased pro-apoptotic gene expression and disturbances in cytokines synthesis may account for the proliferation of Tregs and promote tumorigenesis and metastasis. In conclusion, our study provides a new insight into understanding of peripheral Tregs accumulation in aged malignancies.

## Materials and Methods

### Ethics Statement

All participants signed a statement of written informed consent. The procedures described in this study were approved by the ethics committee of the Second Hospital of Anhui Medical University.

### Patients and Healthy Donors

Peripheral blood (PB) specimens from 38 aged UBC patients (62 to 79 years old, mean age 70.13), 34 aged healthy controls (60 to 74 years old, mean age 68.72) and 37 young healthy controls (22 to 56 years old, mean age 43.25) were examined at Second Hospital of Anhui Medical University from 2010–2013. None of the patients received anticancer therapy before being enrolled. Concurrence of autoimmune disease, human immunodeficiency virus (HIV) and syphilis was excluded for all enrolled individuals. Clinical staging was performed after preoperative radiological examination and by clinical parameters in conjunction with pathological examination of transurethral resection specimens according to the TNM classification of 1997 (Union Internationale Contre le Cancer).

### Antibodies and Flow Cytometry

The following monoclonal antibodies (mAbs) specific for human surface antigens were purchased from Beckman Coulter-Immunotech (Marseille, France): phycoerythrin (PE)-conjugated anti-CD4 antibodies (13B8.2 clone); fluorescein isothiocyanate (FITC)-conjugated anti-CD25 (B1.49.9 clone); Peridinin Chlorophyll Protein-Cychrome5.5 (Percp-Cy5.5) conjugated anti-CD127 (SK3 clone); three color reagent kit for Tregs containing of PE-conjugated anti-CD4, FITC-conjugated anti-CD25, and Percp-Cy5.5-conjugated anti-127 (UCHT1, 13B8.2 and B911 clone); and its isotype control antibody. BU-Annexin V-FITC Apoptosis Detection Kit (Biouniquer Nanjing, China) were used for Treg cellular apoptosis assessment. Cells were analyzed and sorted using Coulter Epics XL flow cytometer (FCM) with system II software and COULTER EPICS ALTRA HyPerSortTM System with expo 32 multicomp Software (Beckman Coulter, Miami, FL, USA).

### Examination of Peripheral CD4+CD25+CD127^low^ Cells

About 2–5 ml of PB was collected. All samples were anticoagulated with heparin and examined within 4 h. About 100 µl of anti-coagulated blood was incubated at 25°C for 15 min with 5 ul FITC-conjugated CD25-specific mAb, 5 ul PE-conjugated CD4-specific mAb, 5 ul Percp-Cy5.5-conjugated anti-127 mAb and their appropriate isotype controls. After incubation, red blood cells were lysed and washed in PBS two times. Stained cells were quickly detected using the FCM and analyzed using system ii software. As described previously [14], the frequency of CD4+CD25+CD127^low^ Tregs was expressed as a percentage of CD4+ T cells by sequential gating on lymphocytes and CD4+ T cells.

### Isolation of CD4+CD25^high^ and CD4+CD25- Cells

About 15–20 ml of PB was collected for cell sorting. Peripheral blood mononuclear cells (PBMCs) were isolated by density gradient separation using CAPPEL LSM Lymphocyte Separation Medium (MP Biomedicals, Solon, OH). Isolated PBMCs were washed 3 times with PBS. About 1×10^7^ PBMCs were surface stained with PE-conjugated anti-CD4 and FITC-conjugated anti-CD25. Then CD4+CD25^high^ and CD4+CD25- cells were sorted using the gates on a Becton Counter flow-cytometric cell-sorter (ALTRA HyPerSortTM System). As we previously described [15], consistent purity of >93% was obtained for both CD4+CD25^high^ T cell and CD4+CD25-cell fractions.

### Cell Culture and Treatment

Flow cytometry sorted CD4+CD25high Tregs and CD4+CD25- Teffs were plated in 24-well plates (Wuxi Nest Biotechnology Co., Ltd, Wuxi, China), and cultured in RPMI-1640 (HyClone) containing 10% FBS (Gibco) and antibiotics for 24h. Then cells were treated with a subcytotoxic concentrations of H_2_O_2_ (100 µM) for 2 hours as a stress inducer. All cells were incubated at 37°C in a humidified 5% CO_2_ atmosphere incubator.

### SiRNA Transfection

Sorted CD4+CD25^high^ and CD4+CD25- cells from aged UBC patients and healthy donors were transfected with siRNA by using lipofectamine 2000 (Invitrogen) according to the manufacturer’s protocols. The final concentration of siRNA was 33 nM. The duplexes of siRNA targeting *SENEX* mRNA (the sequences listed in Tab 1) and control siRNA (scrambled sequence) were synthesized by Invitrogen (Shanghai, China).

**Table 1:** Sequences used for Real-time quantitative PCR primers and SiRNA transfection.

Name	Sequences(5′→3′)
*SENEX* - Forward	TTGCTCTGTTTTCCAGATTGGA
*SENEX* - Reverse	GCCCCAGTGCTTGAGGCT
*FoxP3*- Forward	CTGACCAAGGCT TCATCTGTG
*FoxP3*- Reverse	ACTCTGGGAATGTGC TGTTTC
*Caspase-3*- Forward	TAGTGTGTGTGTTGCTCAGTC
*Caspase-3*- Reverse	CTCGACAAGCCTGAATAAAG
*P53*- Forward	CCCGGATGGAGATAACTTG
*P53*- Reverse	CACAGTTGTCCATTCAGCAC
*P16-* Forward	TCTGAGCTTTGGAAGCTCTCA
*P16*- Reverse	GAGAACTCAAGAAGGAAATTGG
*P21*- Reverse	ATGCAGCTCCAGACAGATGA
*P2* - Forward	CGCAAACAGACCAACATCAC
*GAPDH-* Reverse	TGCACCACCAACTGCTTAGC
*GAPDH-* Forward	GGCATGGACTGTGGTCATGAG
SiRNA-*SENEX*-homo-236	GCACCACCAUCAAAGUUAUTT
	AUAACUUUGAUGGUGGUGCTT
SiRNA-*SENEX*-homo-1189	GGAGCUGCCAUUAGAAUCATT
	UGAUUCUAAUGGCAGCUCCTT
SiRNA-*SENEX*-homo-436	GCCGGUUUAUCCAAUCUCUTT
	AGAGAUUGGAUAAACCGGCTT

### Examination of Cellular Apoptosis

Flow cytometry sorted CD4+CD25^high^ Tregs and CD4+CD25- Teffs were stained with Annexin V-fluorescein isothiocyanate (Annexin V-FITC) and propidium iodide (PI, Biouniquer, Nanjing, China) according to the manufacturer’s instructions. Cells were then incubated for 15 min in dark before being subject to analysis on a flow cytometer (FACSCalibur, Becton Dickinson).

### Real-time Quantitative PCR Analysis

Total RNA was isolated using RNeasy Kit (Qiagen). cDNA was synthesized with the SuperScript III (Invitrogen) reverse transcriptase synthesis kit. Quantitative real time PCR was performed on the 7000 Real-Time PCR System (Applied Biosystems) using the Mesagreen qPCR Master Mix Plus for SYBR Assay (Takara). The primers used and their sequences are listed in Table 1.

### Serum Cytokines

Serum samples from UBC patients and healthy controls were collected. The concentration of IL-2, IL-10, IL-17, TNF-α and TGF-β was determined using enzyme-linked immunosorbent assay (ELISA) kits (eBioscience) according to the manufacturer’s instructions.

### Statistical Analyses

Statistical analysis was performed using the SPSS 15.0 software (SPSS Inc., Chicago, IL). Statistical significance of differences was determined by the non parametric unpaired Mann-Whitney *U*-test and the parametric Student’s *t* test for paired or unpaired samples when appropriate. Correlations were evaluated using the Pearson’s coefficient test. Differences were considered significant for p-values less than 0.05.

## Results and Discussion

### Markedly Increased CD4+CD25+CD127^low^ Treg Frequency and Transcription Factor FoxP3 mRNA Expression in Aged UBC Patients

**Figure 1:**
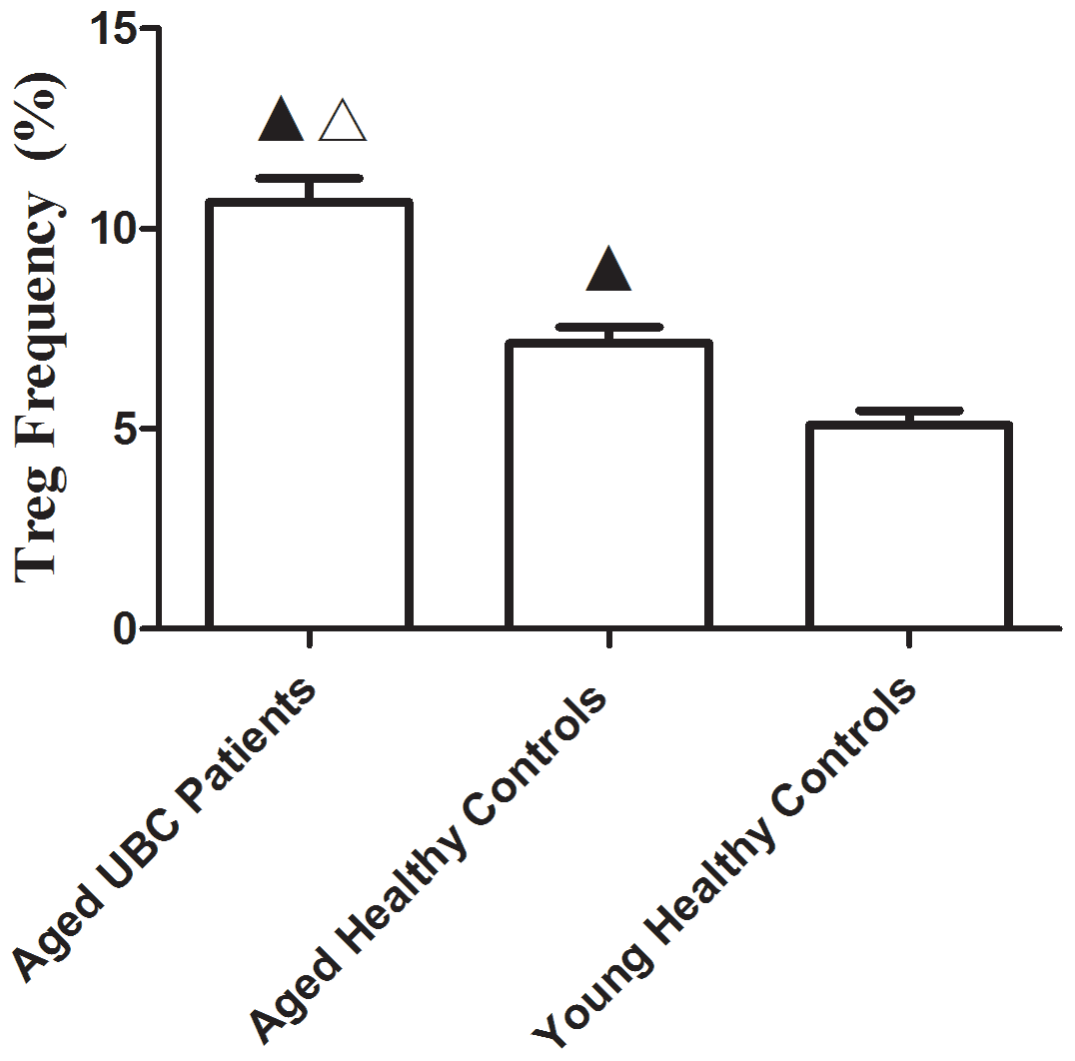
CD4+CD25+CD127^low^ Treg frequency significantly increased in aged UBC patients. Treg frequency was detected using Flow cytometry. An increased CD4+CD25+127^low^ Tregs frequency (10.67±0.58%) was observed in Aged UBC Patients as compared to Aged Healthy Controls (7.14±0.41%) and Young Healthy Controls (5.09±0.37%). Expressed as the mean ± SD, data were analyzed with the parametric Student’s t test. ▴ vs. Young Healthy Controls, P<0.05; △vs. Aged Healthy Controls, P<0.05.

In view of the critical role of peripheral Tregs accumulation in aged malignancies, the CD4+CD25+CD127^low^ Treg frequency was examined in peripheral blood of aged UBC patients, aged healthy controls and young healthy controls. In accordance with previous studies, an increase in Treg frequency was observed in both aged UBC patients and aged healthy controls, as compared to young healthy controls. Meanwhile, Tregs frequency was significantly higher in aged UBC patients than in aged controls (Figure 1). Increased Tregs frequency contributes to age-related immunosuppression, increases the incidence of infection and cancer, and causes morbidity and mortality in the elderly [4]–[6]. One study showed an age-dependent defective tumor clearance, which was correlated with Tregs elevation [16]. Importantly, CD25-depletion led to tumor clearance, suggesting that aged Tregs limit anti-tumor immunity [16].

**Figure 2:**
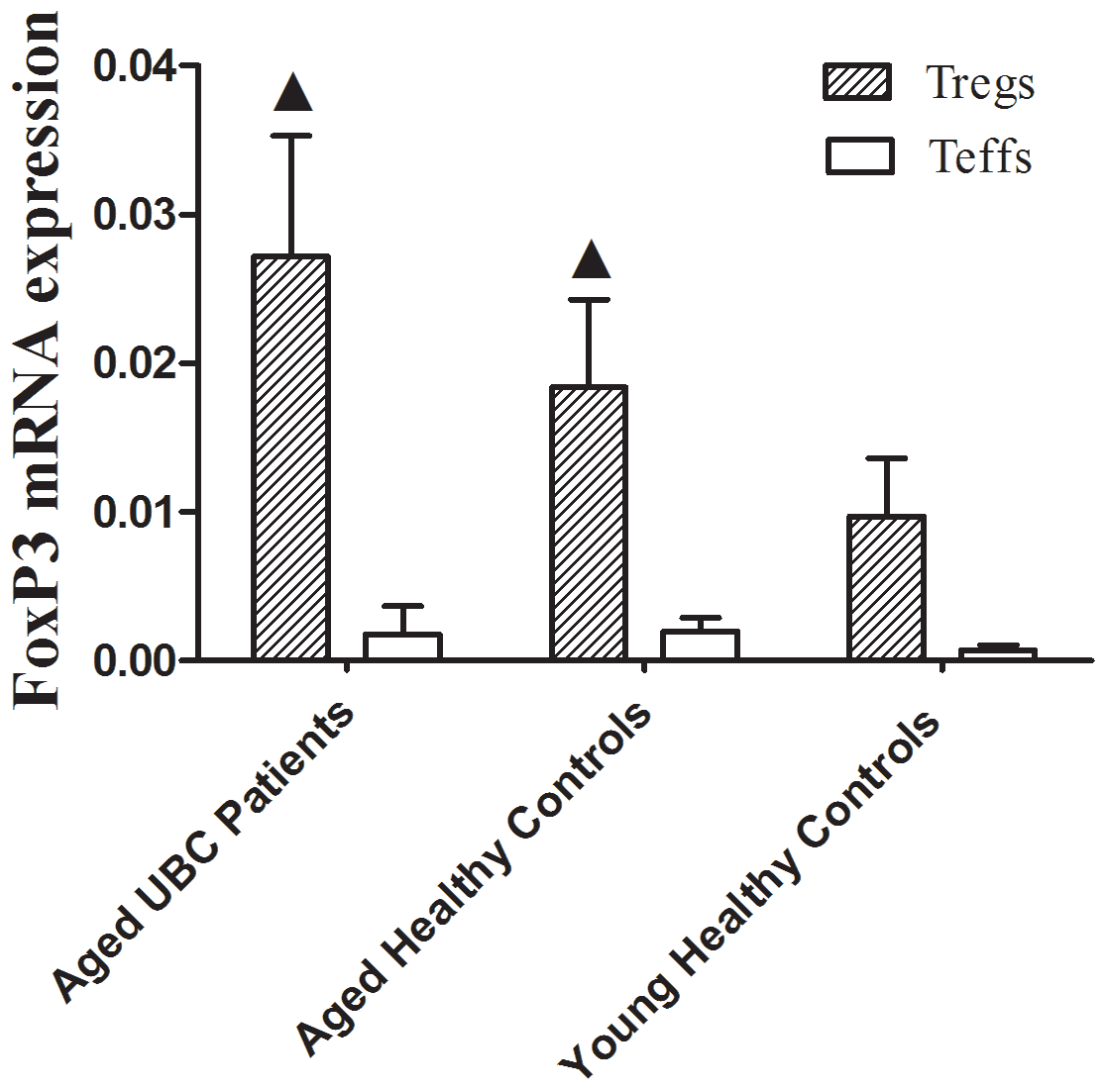
FoxP3 mRNA expression significantly increased in Tregs sorted from aged UBC patients. Tregs were sorted by FACS, FoxP3 mRNA level was detected by real-time quantitative PCR. FoxP3 mRNA expression was increased in Tregs of both Aged UBC patients (2^−△△CT^ = 0.0272±0.0081) and Aged Healthy Controls (0.0184±0.0059) as compared to Young Healthy Controls (0.0097±0.0039) (p = 0.035, 0.021 respectively). No difference was detected in Tregs FoxP3 mRNA expression between Aged UBC Patients and Aged Healthy controls. Expressed as the mean ± SD, the date were analyzed with the Mann-Whitney *U*-test. ▴ vs. Tregs sorted from Young Healthy Controls, P<0.05.

The transcription factor FoxP3 plays a vital role in the maintenance of self-tolerance and high FoxP3 expression is required for suppressive function in human CD4+Tregs [17]–[19]. In this study, FoxP3 mRNA expression in both aged UBC patients and healthy individuals was examined. As expected, FoxP3 mRNA levels significantly increased in CD4+CD25^hi^ Tregs sorted from aged UBC patients and aged healthy controls, as compared to young healthy controls. However, there was no significant difference in FoxP3 mRNA expression between Tregs from aged UBC patients and aged healthy controls (Figure 2). The discrepancy between Tregs frequency and Foxp3 mRNA expression may have been caused by functional differences, as previous studies demostrated that FoxP3+ T cells in humans are functionally heterogeneous [1]. Upregulated FoxP3 mRNA expression, together with the increased Tregs frequency, may increase tumors escape, immune suppression and lead to ineffective tumor clearance in aged UBC patients.

### Upregulated *SENEX* mRNA Expression and Downregulated Gene Expression of *P53*, *P16*, *P21* and *Caspase-3* in CD4+CD25^hi^ Tregs of Aged UBC Patients

**Figure 3:**
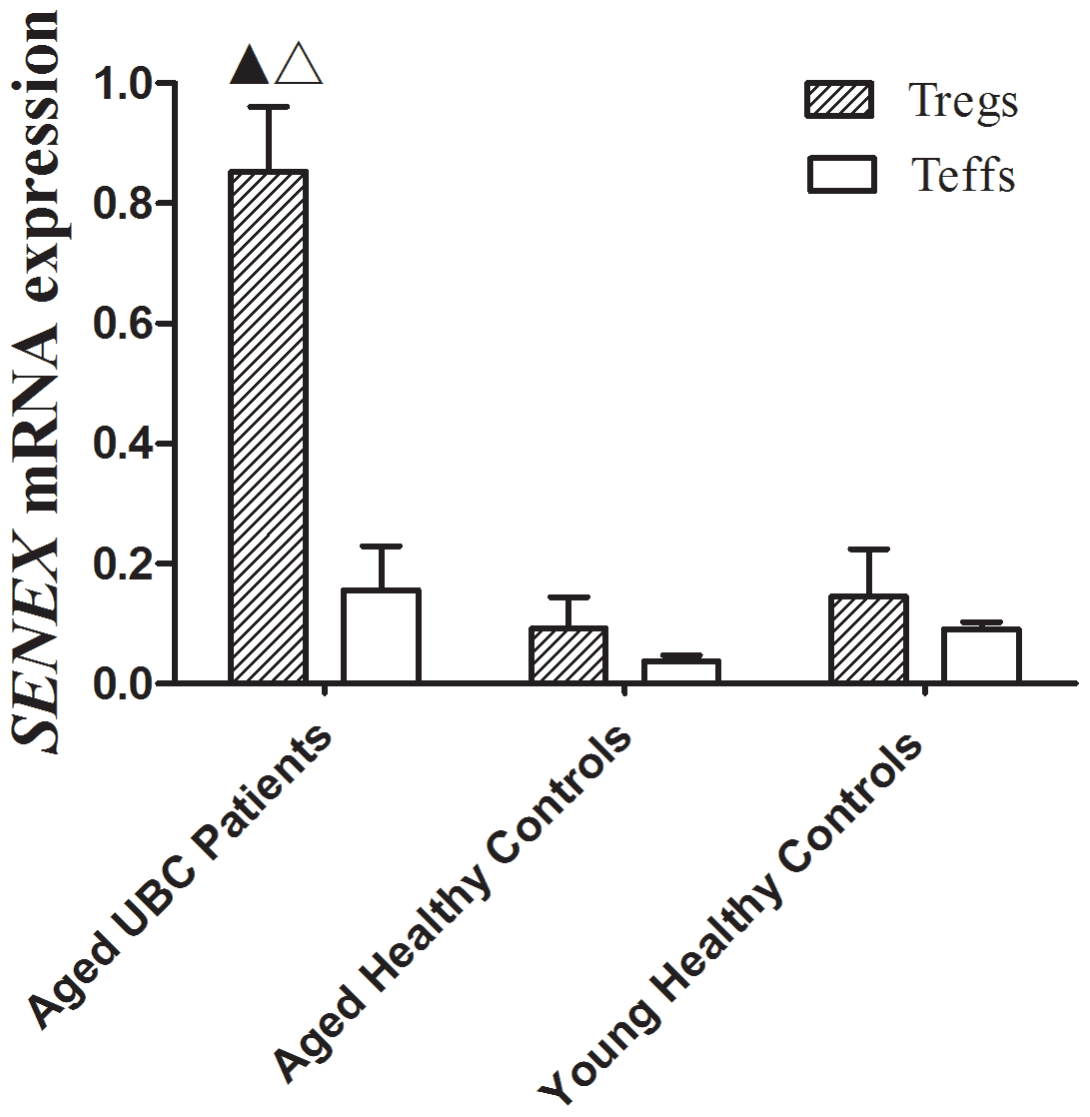
Upregulated *SENEX* mRNA expression in CD4+CD25^hi^ Tregs sorted from Aged UBC patients. Tregs were sorted by FACS, *SENEX* mRNA level was detected by real-time quantitative PCR. An significant increase in *SENEX* mRNA expression was detected in CD4+CD25^hi^ Tregs (2^−△△CT^ = 0.8532±0.1078) as compared to CD4+CD25- Teffs (0.1564±0.0731) in Aged UBC patients (*p* = 0.027), and this increase was not observed in other groups. Tregs *SENEX* mRNA expression in Aged UBC patients was signicantly higher than those in Aged Healthy Controls (0.0923±0.0519) and Young Healthy Controls (0.1455±0.0792) (p<0.05 for each Mann-Whitney *U*-test). Data were expressed as the mean ± SD. ▴ vs. Teffs sorted from Aged UBC patients, P<0.05; △vs. Tregs in Aged Healthy Controls and Young Healthy Controls, P<0.05.

**Figure 4:**
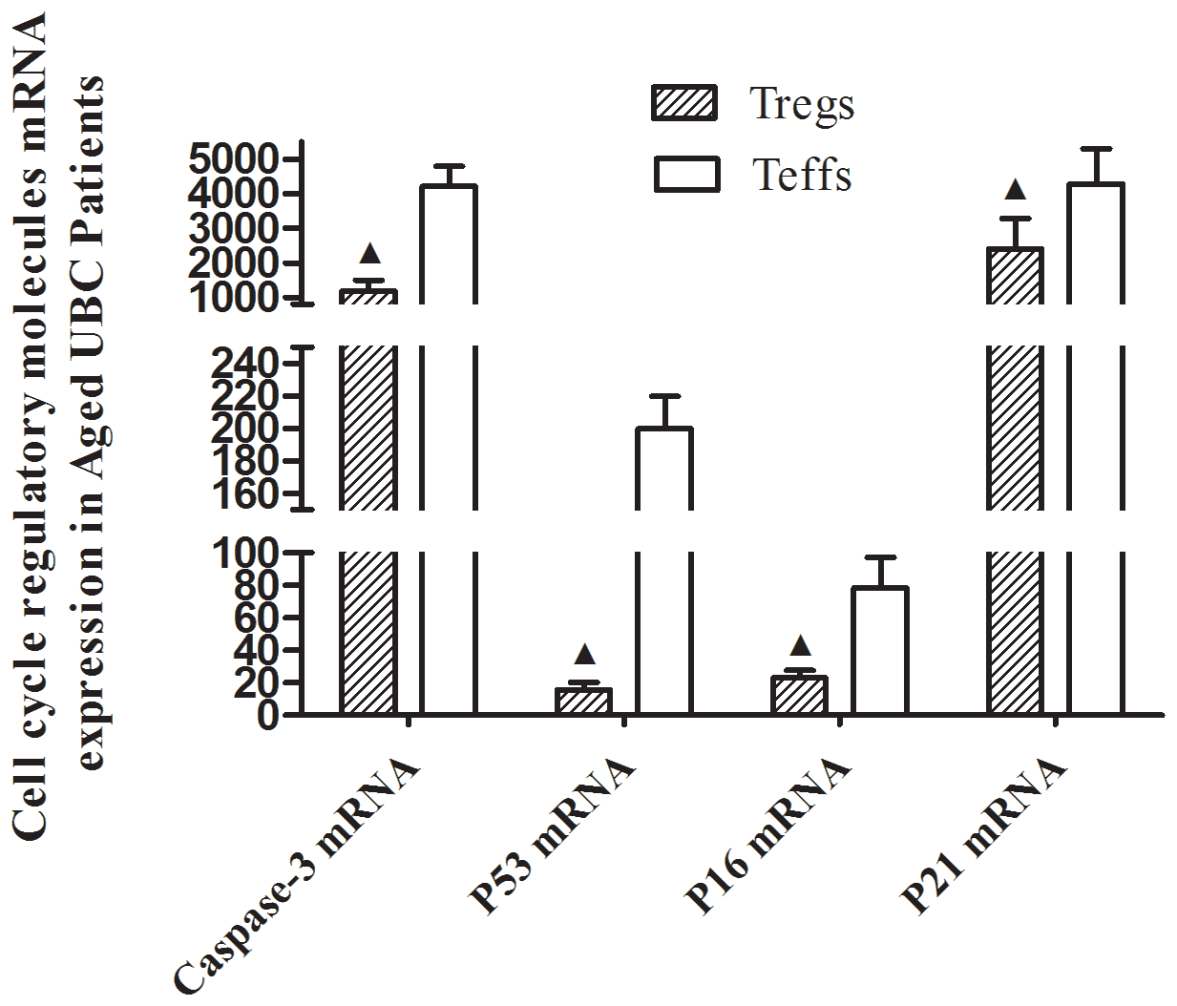
Downregulated gene expression of *P53*, *P16*, *P21* and *Caspase-3* in CD4+CD25^hi^ Tregs of aged UBC patients. Tregs were sorted by FACS, mRNA levels were detected by real-time quantitative PCR. In Aged UBC Patiens, apoptosis regulatory genes Caspase-3, P53, P16 and P21 mRNA expression (2^−△△CT^ = 1183.42±321.17, 15.66±4.83, 23.06±4.59 and 2415.85±863.72, respectively) decreased in Tregs of Aged UBC patients (*p*<0.05 for each Mann-Whitney U Test). Data were expressed as the mean ± SD. ▴ vs. Teffs sorted from Aged UBC patients, P<0.05.

To evaluate the potential contribution to peripheral Tregs accumulation, *SENEX* mRNA expression of CD4+CD25^hi^ Tregs and CD4+CD25- effector T (Teff) cells was examined. Significant increase in *SENEX* mRNA expression was detected in CD4+CD25^hi^ Tregs as compared to CD4+CD25- Teffs in aged UBC patients, and this increase was not observed in other groups. Meanwhile, *SENEX* mRNA expression of Tregs in aged UBC patients was signicantly higher than those in aged and young healthy controls (Figure 3). Furthermore, the mRNA levels of apoptosis regulatory gene Caspase-3, P53, P16 and P21 (2^−△△CT^ = 1183.42±321.17, 15.66±4.83, 23.06±4.59 and 2415.85±863.72, respectively) were significantly decreased in CD4+CD25 ^hi^ Tregs sorted from aged UBC patients, as compared to CD4+CD25- Teffs (Figure 4). No significant differences of apoptosis regulatory gene expression were detected in CD4+CD25 ^hi^ Tregs sorted from aged and young healthy controls, as compared to Teffs.

*SENEX* gene has been proved to provide a unique gatekeeper function in the SIPS and apoptosis pathways in ECs [12]. Over expression of *SENEX* gene could cause ECs senescence, and these *SENEX*-induced senescent cells were resistant to tumor necrosis factor (TNF-α) induced apoptosis when ECs were exposed to subcytotoxic concentrations of H_2_O_2_. Furthermore, *SENEX* overexpression induced an increase in both the mRNA and protein levels for p16 and there was a decrease in the protein expression of the hyperphosphorylated Rb. These results indicated that *SENEX* activated the p16/pRb pathway [12]. Although the critical role of *SENEX* gene in protecting ECs against cellular apoptosis has been well demonstrated, the expression of *SENEX* gene in Tregs remains largely unknown. In the present study, *SENEX* gene mRNA expression was found to be significantly upregulated in peripheral CD4+CD25^hi^ Tregs sorted from aged UBC patients, as compared to Teffs. The up-regulated *SENEX* gene may play a similar role of inducing Tregs senescenc and protecting them from cellular apoptosis. Previous studies have shown that senescent growth arrest is established and maintained by the p53/p21 and/or p16/pRB tumor suppressor pathways [20]–[22]. It is known that wild-type p53 protein can induce cell apoptosis [23]. Caspase 3 is a downstream effector cysteine protease in the apoptotic pathway, and it is regarded as one of the main executors of apoptosis [24]. P16 gene and P21 gene can inhibit cyclin-dependent kinases (CDK) and induce cell cycle arrest at G0–G1 phase [25]–[26]. We reasoned that cell cycle regulatory molecules, including p53, p21, and p16 might be involved in Tregs accumulation in aged UBC patients. Our results indicated that the expression of apoptosis regulatory gene *P53*, *P16*, *P21* and *Caspase-3*, which could promote cellular apoptosis as previously described [23]–[26], downregulated in Tregs of aged UBC patients. The up-regulated *SENEX* gene together with down-regulated pro-apoptotic gene may lead to decreased apoptosis in Tregs, and Tregs accumulation in the development and progression of aged urinary bladder cancer.

### Increased Levels of TGF-β1, IL-10, IL-17 and TNF-α Serum Concentration and Decreased Levels of IL-2 Serum Concentration in Aged UBC Patients

**Figure 5:**
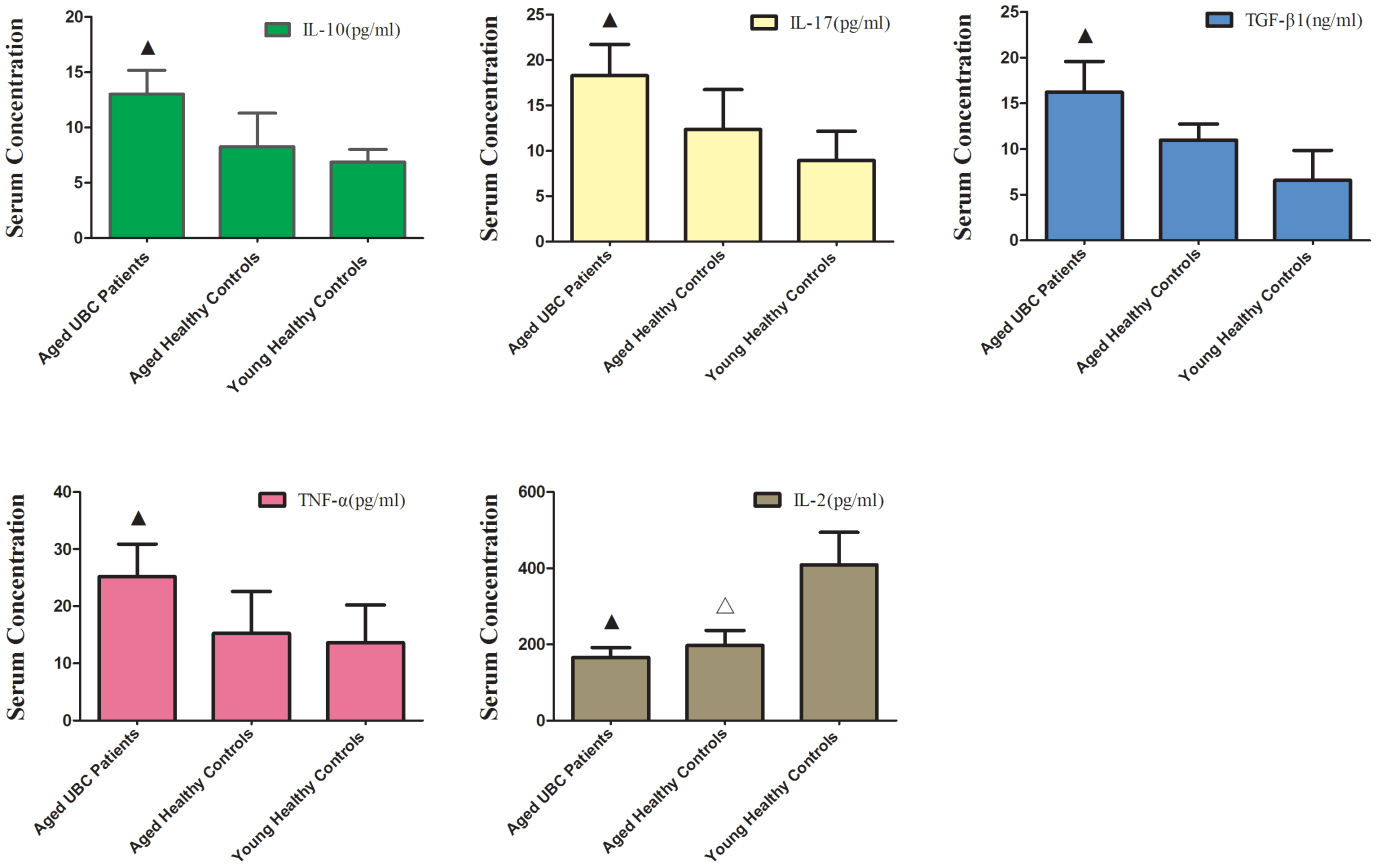
Increased levels of TGF-β1, IL-10, IL-17 and TNF-α serum concentration and decreased levels of IL-2 serum concentration in aged UBC patients. Expressed as the mean ± SD, data were analyzed with the parametric Student’s T test. ▴ vs. Aged Healthy Controls and Young Healthy Controls, P<0.05; △vs. Young Healthy Controls, P<0.05.

For an indirect assessment for T cell function, we evaluated serum cytokine profiles related to tumor immunopathology. Compared with aged healthy controls and young healthy controls (*p*<0.05 for each T Test), there was an obvious increase in concentration of IL-10 (13.03±2.16 pg/ml), IL-17 (18.29±3.45 pg/ml), TGF-β1 (16.22±3.35 ng/ml) and TNF-α (25.18±5.74 pg/ml) in serum of aged UBC patients. Meanwhile, there was a decrease in IL-2 serum concentration both in aged UBC patients (165.29±26.45 pg/ml) and aged healthy controls (196.69±32.38 pg/ml), as,compared to young healthy controls (409.24±90.81). However, no statistically significant difference in IL-2 serum concentration was observed between aged UBC patients and aged healthy controls (p = 0.243) (Figure 5).

As the primary source of TGF-β1 and IL-10 in the maintenance of self-tolerance and T-cell homeostasis, regulatory T cells inhibit effector T cells primarily through the production of cytokines, such as TGF-β1 and IL-10 [27]. This study shows that serum concentrations of both TGF-β1 and IL-10 significantly increased in aged UBC patients. Elevated levels of TGF-β1 and IL-10 suggest that the accumulated Tregs in aged UBC patients may possess high suppressive capacity, inhibit antitumor immune responses and promote tumor immune escape. TNF-α is the prototypical proinflammatory cytokine. Although originally shown to be toxic to tumor cells in high doses, TNF-α has shown to have the tumor-promoting function [28]. Likewise, Cytokine IL-17, secreted by Th17 cells, is generally preferable in the generation of T-cell anti-tumor immunity, but these effects are eclipsed by their roles in the growth of tumors [29]. In the present study, high levels of TNF-α and IL-17 in aged UBC patients may promote tumorigenesis, as previous study had shown that they could promote tumor angiogenesis.

Interleukin-2 is produced mainly by CD4+ T helper cells and CD8+ T cells in secondary lymphoid organs, and IL-2 production is strongly induced following activation by antigen [30]. Able to activate multiple immune-cell subsets, including T cells, interleukin-2 is essential for the development, survival, and function of Treg cells [30]–[31]. Substantial decline in IL-2 with age has been described, mainly based on ex vivo assays, in which T cell production of IL-2 appears to be deficient [6]. IL-2 acts on cells expressing either the high-affinity trimeric IL-2R or the low-affinity dimeric IL-2R. High levels of the trimeric IL-2R are transiently expressed by CD4+ and CD8+ T cells following TCR activation whileTreg cells constitutively express high levels of rimeric IL-2R [30]. In our study, an obvious decline in IL-2 serum concentration both in aged UBC patients and aged healthy controls was observed. Low concentrations of IL-2 may preferentially stimulate the proliferation of Treg cells through the high-affinity trimeric IL-2R, and contribute to peripheral Tregs accumulation in the elderly population.

### *SENEX* Expression was Correlated with P53,TGF-β1 and IL-2 expression in Aged UBC Patients

**Figure 6:**
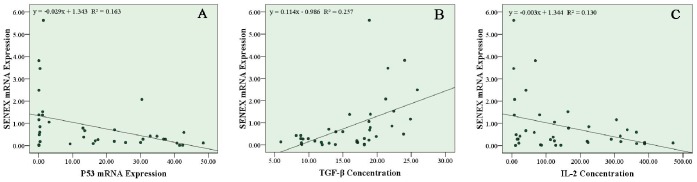
*SENEX* mRNA expression was correlated with P53,TGF-β1 and IL-2 expression in Aged UBC Patients. Using Pearson’s coefficient test, *SENEX* expression was negatively correlated with (**A**) P53 mRNA expression (Pearson correlation coefficient = -0.405, p = 0.012) and (**B**) IL-2 concentration (Pearson correlation coefficient = -0.362, p = 0.026) in Aged UBC Patients. (**C**) Positive correlation between *SENEX* expression and TGF-β1 concentration (Pearson correlation coefficient = 0.507, p = 0.001) was figured out by Pearson’s coefficient test.

We further explored the potential relationship between senescence gene *SENEX* mRNA level and serum cytokine profiles or apoptosis regulatory gene mRNA expression. Using the Pearson correlation analysis, our data indicated that *SENEX* mRNA expression was negatively correlated with P53 mRNA expression (p = 0.012) and IL-2 concentration (p = 0.026), while a positive correlation between *SENEX* mRNA expression and TGF-β1 concentration (p = 0.001) was figured out in Aged UBC Patients. *SENEX* gene expression was negatively correlated with *P53* mRNA expression (Figure 6A), implying that *SENEX* gene may provide its special function by down-regulating the pro-apoptotic gene *P53* expression. Furthermore, *SENEX* gene expression was positively correlated with TGF-β1 level (Figure 6B), suggesting that the up-regulated expression of *SENEX* gene may account for the high suppressive capacity of accumulated Tregs in aged UBC patients. Finally, *SENEX* gene was negatively correlated with IL-2 serum concentration (Figure 6C). The proliferation of Tregs may highly suppress the CD4+ T helper cells and CD8+ T cells, and inhibit the secretion of IL-2 in aged UBC patients.

### Silencing Down *SENEX* Gene Expression Increased Tregs Apoptosis in Response to H_2_O_2_-mediated Stress In vitro

Our above data suggested that the upregulated SENEX gene expression was negatively correlated with *P53* mRNA expression in CD4+CD25^high^ Tregs, which implied that *SENEX* gene may inhibit Tregs cellular apoptosis by down-regulating the pro-apoptotic gene expression. To investigate whether Tregs from aged UBC patients have a lower ratio of cell apoptosis, CD4+CD25^high^ Tregs and CD4+CD25- Teffs were sorted, followed by flow cytometric analysis after being stained with Annexin V-FITC and PI. Annexin V specifically binds to the exposed phosphatidylserine on the apoptotic cell surface while PI can penetrate into dead cells and intercalates with nucleic acid [32]. Our data reported that Tregs from aged UBC patients had fewer Annexin V+ apoptotic cells (0.84±0.34%) as compared to Teffs(9.27±3.66%) (Figure S1). This is consistent with the previous study [11], suggesting that Tregs from aged UBC patients are more resistant to apoptosis.

**Figure 7:**
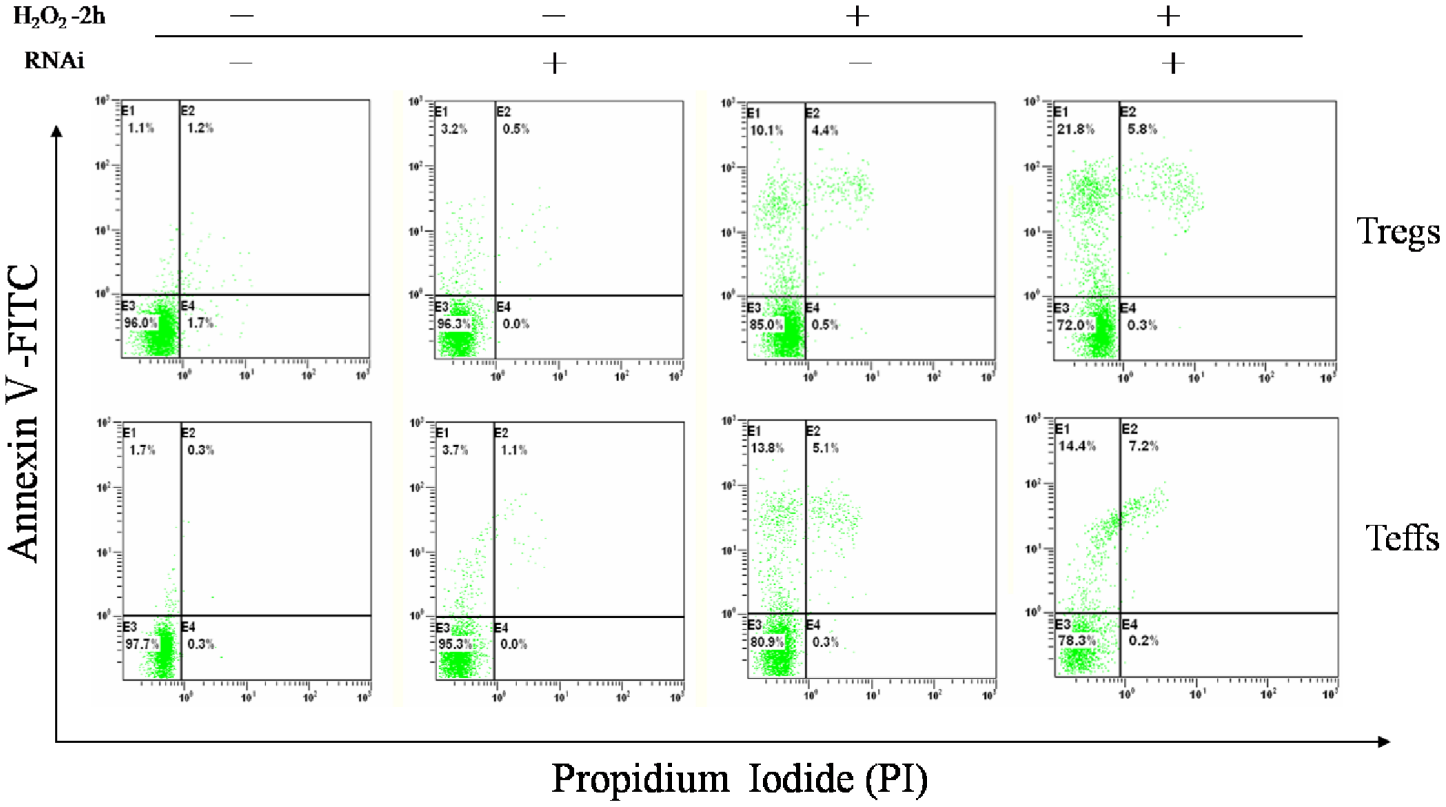
Silencing down *SENEX* gene expression increased Tregs apoptosis in response to H_2_O_2_-mediated stress in vitro. Sorted CD4+CD25^high^ Tregs were stained with Annexin V-FITC before Flow cytometric analysis. Cell apoptosis were detected as Annexin V+ apoptotic cells. 100 µM H_2_O_2_ treatment for 2 hours increased apoptotic cells in both Tregs (11.2±2.6%) and Teffs (13.1±4.3%), with no significant difference observed in H_2_O_2_ induced apoptosis between Tregs and Teffs. Silencing down *SENEX* gene with RNA interference (RNAi) for 24h, follwed by H_2_O_2_ treatment for 2 hours, increased apoptotic cells in Tregs (21.5±3.4%), but undergoing the same procedures, apoptotic cells wasn’t found to increase in Teffs (13.9±3.1%). No difference was observed in cellular apoptosis between Tregs and Teffs when cells were treated with SiRNA transfection alone.

**Figure 8:**
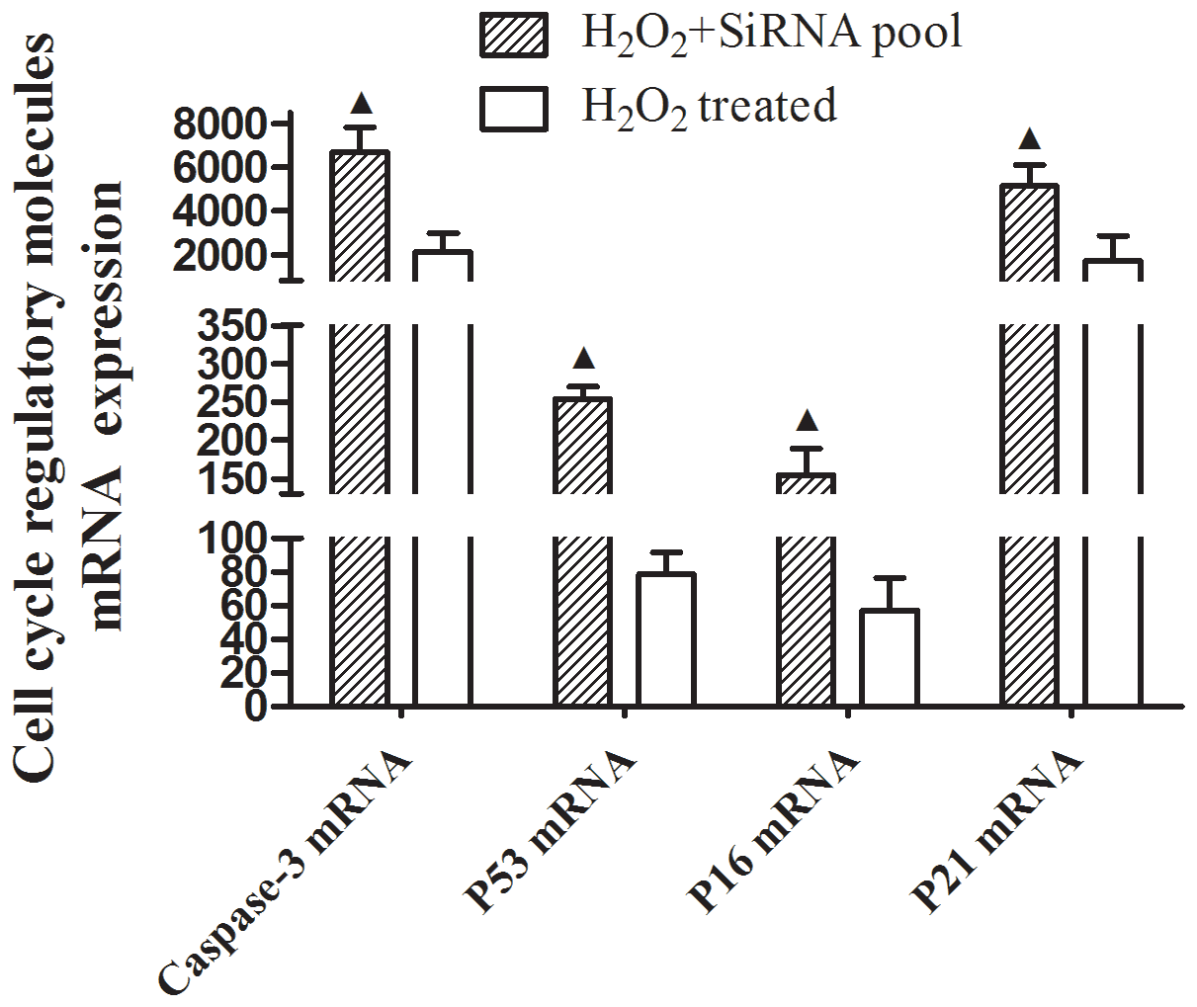
Silencing down Tregs *SENEX* gene led to upregulation of *P53*, *P16*, *P21* and *Caspase-3* mRNA expression in response to stress. Gene mRNA levels were detected by real-time quantitative PCR. After *SENEX* gene was silenced down for 24 hours, followed by H_2_O_2_ treatment for 2 hours, apoptosis regulatory genes *Caspase-3*, *P53*, *P1*6 and *P21* mRNA expression (2^−△△CT^ = 6705.73±1124.07, 253.08±16.01, 154.58±35.12 and 5135.79±985.47, respectively) upregulated in CD4+CD25^hi^ Tregs (*p*<0.05 for each Mann-Whitney U Test). Data are expressed as the mean ± SD. ▴ vs. H_2_O_2_-treated cells, P<0.05.

To further study the potential role of *SENEX* gene in Tregs apoptosis in aged UBC patients, we silenced down *SENEX* gene expression with RNA interference in short-term cultured Tregs in response to H_2_O_2_ induced oxidative stress. H_2_O_2_ is a known inducer of oxidative stress when delivered in a subcytotoxic dose [33]. In the present study, sorted CD4+CD25^high^ Tregs and CD4+CD25- Teffs were exposed to subcytotoxic concentrations of H_2_O_2_ (100 µM) for 2 hours. Our results indicated that 100 µM H_2_O_2_ treatment increased Annexin V+ apoptotic cells in both CD4+CD25^high^ Tregs (11.2±2.6%) and CD4+CD25- Teffs (13.1±4.3%), and no significant difference was observed in H_2_O_2_ induced cellular apoptosis between Tregs and Teffs. Silencing down *SENEX* gene by SiRNA pool transfection for 24h, follwed by 100 µM H_2_O_2_ treatment for 2 hours, increased Annexin V+ apoptotic cells in CD4+CD25^high^ Tregs (21.5±3.4%), but undergoing the same procedures, Annexin V+ apoptotic cells were not found to increase in CD4+CD25-Teffs (13.9±3.1%) (Figure 7). Moreover, no significant difference was observed in cellular apoptosis in both Tregs and Teffs when cells were only treated with SiRNA transfection without H_2_O_2_ treatment (Figure 7). Furthermore, silencing down Tregs *SENEX* gene expression led to upregulation of pro-apoptotic gene *P53*, *P16*, *P21* and *Caspase-3* mRNA expression in response to stress (Figure 8). The transfection efficiency was 45±7.2%, which was confirmed by fluorescence-labeled FAM-SiRNA. The *SENEX* mRNA expression inhibition rate was more than 90% as compared to noncoding RNA (ncRNA) (Figure S2).

Previous study demonstrated that *SENEX* gene contributed to the apoptosis resistance of human ECs [12]. In the present study, silencing down *SENEX* gene expression increased Tregs apoptosis in response to H_2_O_2_ induced oxidative stress. Our data suggested that *SENEX* gene may play a similar role of protecting Tregs from oxidative stress induced cellular apoptosis in aged UBC patients. Furthermore, exclusively silencing down *SENEX* gene expression did not increase apoptotic Tregs. This implies that *SENEX* may play its unique role by a stress-dependent pathway, and *SENEX* gene expression may be initialed by oxidative stress. In addition, silence down *SENEX* gene expression increased pro-apoptotic gene *P53*, *P16*, *P21* and *Caspase-3* expression, which illustrated that *SENEX* gene expression affected pro-apoptotic gene expression, and subsequently affected Tregs cellular survival in aged UBC patients.

## Conclusion

In the present study, we found that senescence gene *SENEX* mRNA expression significantly upregulated in peripheral Tregs in aged UBC patients. Silencing down *SENEX* gene expression increased Tregs apoptosis in response to H_2_O_2_-mediated stress. *SENEX* gene may protect Tregs from cellular apoptosis through the decreased expression of tumor suppressor P53 gene. These altered expression in transcriptional levels and cytokines may contribute to the increase in Treg proportion during the development and progression of aged urinary bladder cancer, and cellular senescence maybe also involved in this process. In conclusion, our study provides a potential therapeutic target in reducing Treg accumulation during the tumorigenesis in aged urinary bladder cancer patients, even though the detailed mechanisms of Treg accumulation attributed to T cell senesence need to be further explored.

## Supporting Information

Figure S1 **There was a significant decrease in CD4+CD25^high^ Tregs apoptosis in aged UBC patients.** Sorted CD4+CD25^high^ Treg were stained with Annexin V-FITC before Flow cytometric analysis. Cell apoptosis were detected as Annexin V+ apoptotic cells. There was an obvious decrease in Treg cell apoptosis in both Aged UBC Patients (0.84±0.34%) and Aged Healthy Controls (0.60±0.29%) as compared to Teffs. Data are expressed as the mean ± SD and were analyzed with the parametric Student’s t test. ▴ vs. Teffs, P<0.05.
(TIF)

Figure S2 **The** ***SENEX*** **mRNA expression inhibition rate was more than 80%.** Tregs and Teffs were cultured in Opti-MEM 24 hours before SiRNA transfection, and cells were harvested 24 hours after SiRNA transfection/Mock transfection *SENEX* mRNA level was detected by real-time quantitative PCR. Mock transfection was performed using lipofectamine 2000 without any SiRNA. All cell culture experiments were repeated for 3 times.
(TIF)
