# Supplementary figures and images for: A Novel Cellular Senescence Gene, SENEX, Is Involved in Peripheral Regulatory T Cells Accumulation in Aged Urinary Bladder Cancer
Source: PLoS One. 2014 Feb 5;9(2):e87774. doi: 10.1371/journal.pone.0087774 (PMC3914842; doi:10.1371/journal.pone.0087774)

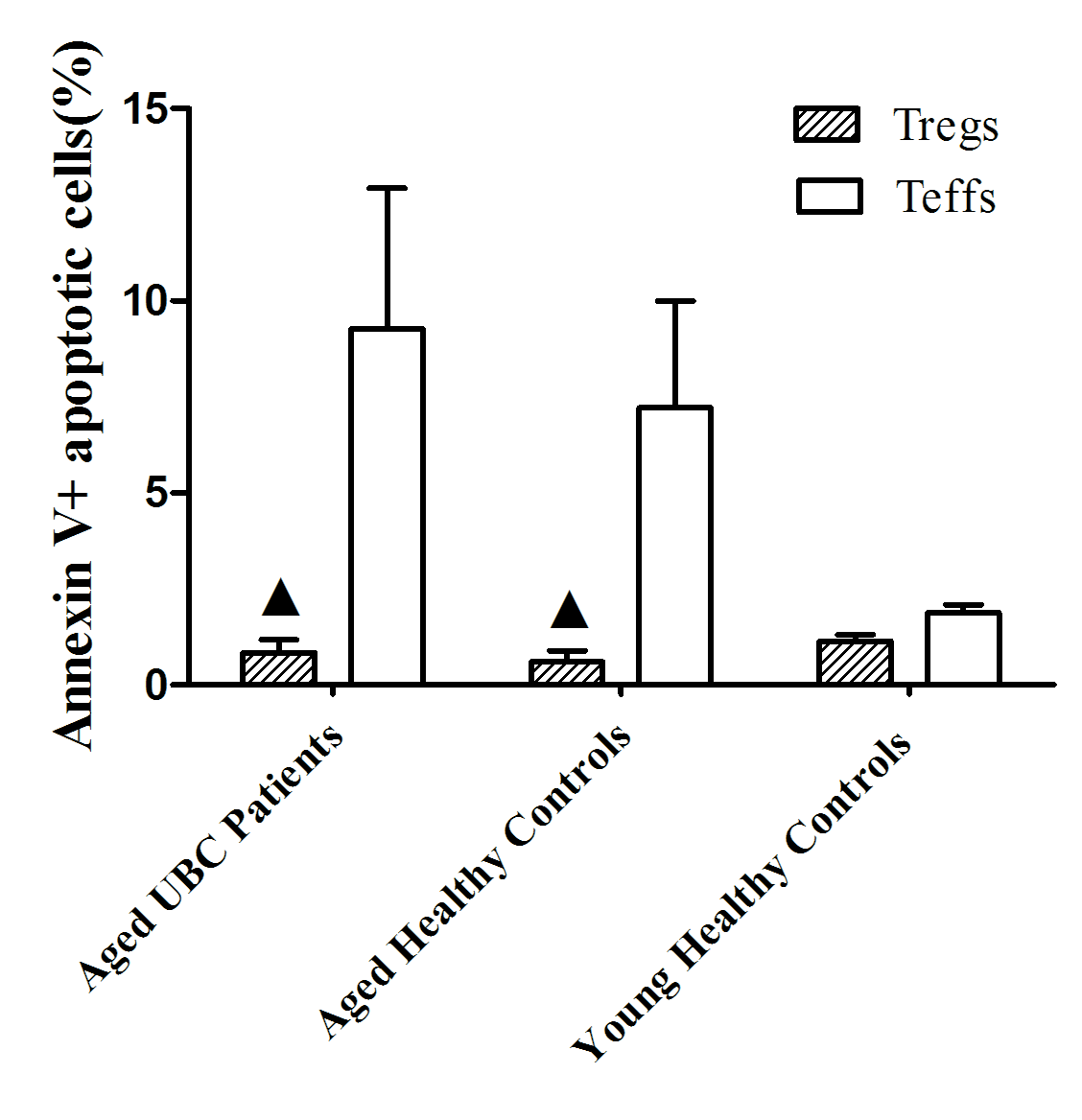

Supplement: Figure S1 — There was a significant decrease in CD4+CD25high Tregs apoptosis in aged UBC patients. Sorted CD4+CD25high Treg were stained with Annexin V-FITC before Flow cytometric analysis. Cell apoptosis were detected as Annexin V+ apoptotic cells. There was an obvious decrease in Treg cell apoptosis in both Aged UBC Patients (0.84±0.34%) and Aged Healthy Controls (0.60±0.29%) as compared to Teffs. Data are expressed as the mean ± SD and were analyzed with the parametric Student’s t test. ▴ vs. Teffs, P<0.05. (TIF) [file pone.0087774.s001.tif]

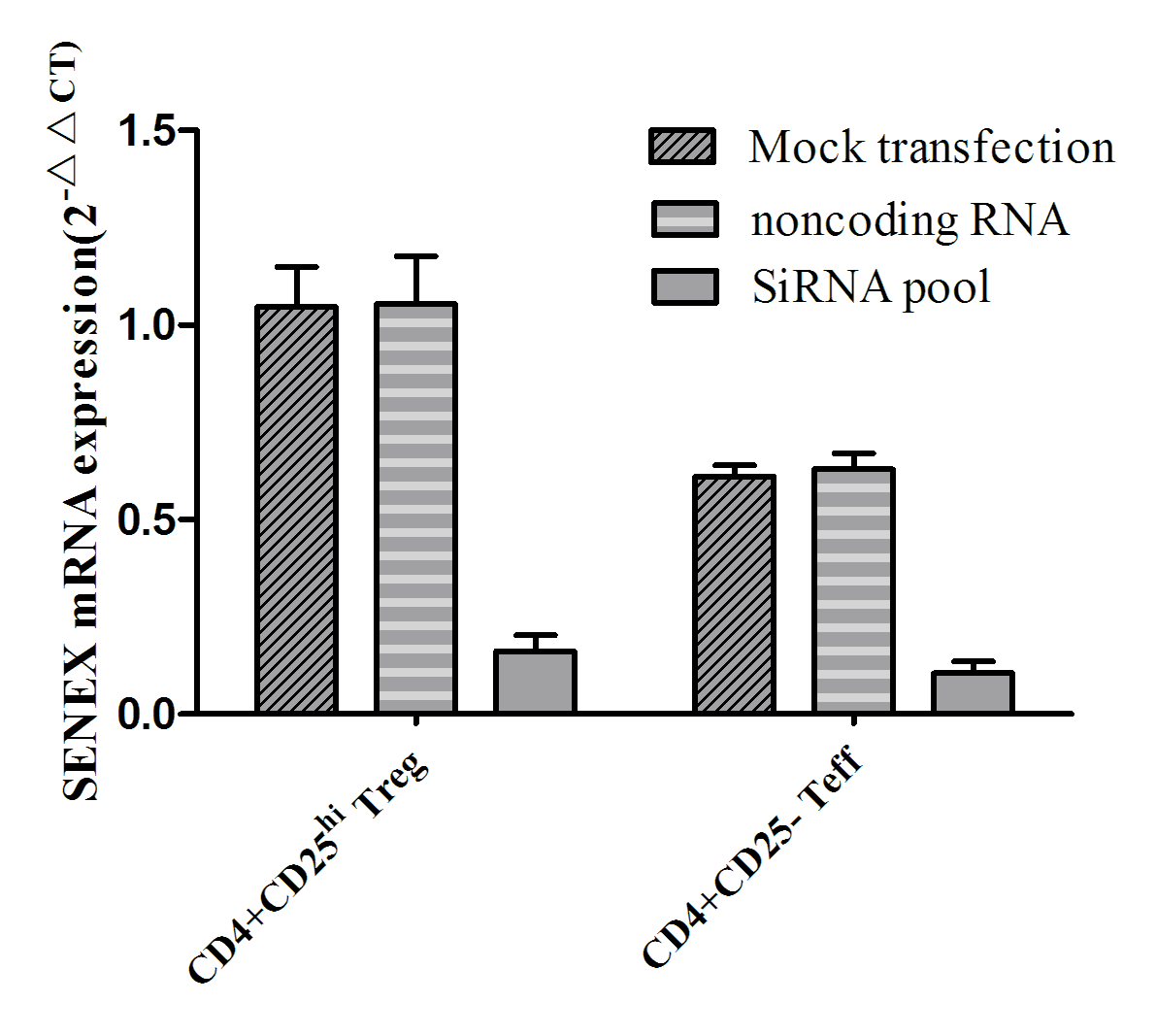

Supplement: Figure S2 — The SENEX mRNA expression inhibition rate was more than 80%. Tregs and Teffs were cultured in Opti-MEM 24 hours before SiRNA transfection, and cells were harvested 24 hours after SiRNA transfection/Mock transfection SENEX mRNA level was detected by real-time quantitative PCR. Mock transfection was performed using lipofectamine 2000 without any SiRNA. All cell culture experiments were repeated for 3 times. (TIF) [file pone.0087774.s002.tif]
